# From chlorite dismutase towards HemQ–the role of the proximal H-bonding network in haeme binding

**DOI:** 10.1042/BSR20150330

**Published:** 2016-03-16

**Authors:** Stefan Hofbauer, Barry D. Howes, Nicola Flego, Katharina F. Pirker, Irene Schaffner, Georg Mlynek, Kristina Djinović-Carugo, Paul G. Furtmüller, Giulietta Smulevich, Christian Obinger

**Affiliations:** * Department for Structural and Computational Biology, Max F. Perutz Laboratories, University of Vienna, A-1030 Vienna, Austria; † Dipartimento di Chimica “Ugo Schiff”, Università di Firenze, Via della Lastruccia 3-13, I-50019 Sesto Fiorentino (FI), Italy; ‡ Department of Chemistry, Division of Biochemistry, BOKU–University of Natural Resources and Life Sciences, Muthgasse 18, A-1190 Vienna, Austria; § Department of Biochemistry, Faculty of Chemistry and Chemical Technology, University of Ljubljana, Aškerčeva 5, 1000 Ljubljana, Slovenia

**Keywords:** chlorite dismutase, electron paramagnetic resonance spectroscopy, H-bonding network, haeme binding, HemQ, resonance Raman spectroscopy

## Abstract

Chlorite dismutase (Cld) and HemQ are structurally and phylogenetically closely related haeme enzymes differing fundamentally in their enzymatic properties. Clds are able to convert chlorite into chloride and dioxygen, whereas HemQ is proposed to be involved in the haeme *b* synthesis of Gram-positive bacteria. A striking difference between these protein families concerns the proximal haeme cavity architecture. The pronounced H-bonding network in Cld, which includes the proximal ligand histidine and fully conserved glutamate and lysine residues, is missing in HemQ. In order to understand the functional consequences of this clearly evident difference, specific hydrogen bonds in Cld from ‘*Candidatus* Nitrospira defluvii’ (NdCld) were disrupted by mutagenesis. The resulting variants (E210A and K141E) were analysed by a broad set of spectroscopic (UV–vis, EPR and resonance Raman), calorimetric and kinetic methods. It is demonstrated that the haeme cavity architecture in these protein families is very susceptible to modification at the proximal site. The observed consequences of such structural variations include a significant decrease in thermal stability and also affinity between haeme *b* and the protein, a partial collapse of the distal cavity accompanied by an increased percentage of low-spin state for the E210A variant, lowered enzymatic activity concomitant with higher susceptibility to self-inactivation. The high-spin (HS) ligand fluoride is shown to exhibit a stabilizing effect and partially restore wild-type Cld structure and function. The data are discussed with respect to known structure–function relationships of Clds and the proposed function of HemQ as a coprohaeme decarboxylase in the last step of haeme biosynthesis in Firmicutes and Actinobacteria.

## Abstract (precis)

Structurally and phylogenetically closely related chlorite dismutase (Cld) and HemQ differ fundamentally in their enzymatic properties. Reconstruction of the proximal H-bonding network renders Cld HemQ-like, the latter being proposed to bind coprohaeme and release protohaeme.

## INTRODUCTION

The peroxidase-chlorite dismutase superfamily (CDE structural superfamily) comprises chlorite dismutases (Clds), chlorite dismutase-like (Cld-like) proteins (HemQs in Gram-positive bacteria) and dye-decolourizing peroxidases (DyPs) [1,2]. Although distinct enzymatic reactions can be assigned to Clds (degradation of chlorite to chloride and dioxygen) and DyPs (H_2_O_2_-mediated one-electron oxidation reactions of various aromatic compounds), knowledge about the biochemical and physiological function of Cld-like proteins is only fragmentary at present. Studies on Cld-like proteins in Gram-positive bacteria suggest an important regulatory role in haeme biosynthesis [3–5]. Consequently, Cld-like proteins in these organisms were renamed HemQ; however, a distinct enzymatic function could not be assigned yet [3–6]. Recently, Dailey and co-workers proposed a novel haeme biosynthesis pathway for Gram-positive bacteria in which HemQ acts as a coprohaeme decarboxylase to yield protohaeme; in this reaction, coprohaeme has to bind to HemQ, react with presumably H_2_O_2_ to release two CO_2_ molecules to yield and release haeme *b*. This finding is also supported by other studies [7–10]. Nevertheless, the mechanism of this reaction is unknown.

**Figure 1:**
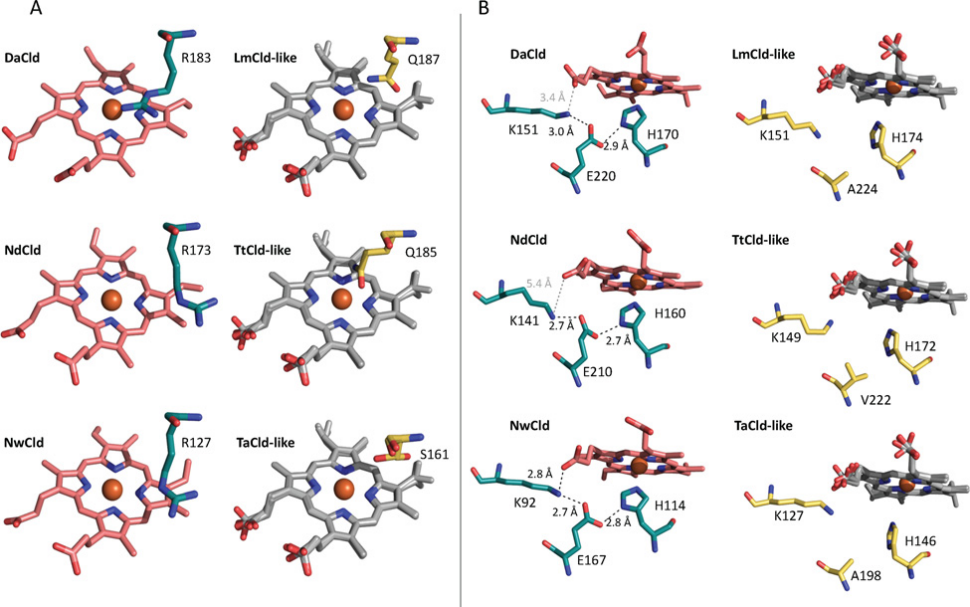
Representative X-ray structures showing the distal (**A**) and proximal (**B**) haeme side in Clds (green with red haeme) and Cld-like proteins (yellow) Since Cld-like structures were solved without the co-factor, haemes (in grey) from Clds were inserted for orientation. Hydrogen bonds are presented as black dotted lines, other distances as grey dotted lines. Structures are depicted of Clds from *D. aromatica* (pdb-code: 3Q08), ‘*Candidatus* Nitrospira defluvii’ (pdb-code: 3NN1) and *N. winogradskyi* (pdb-code: 3QPI), as well as Cld-like structures from *L. monocytogenes* (pdb-code: 4WWS), *T. thermophilus* (pdb-code: 1VDH) and *Thermoplasma acidophilum* (pdb-code: 3DTZ).

Clds and HemQs share the same overall fold and in addition seem to have very similar haeme cavity architecture. In contrast with HemQ, many structural and functional data about Clds have been published in the last decade, which might also contribute to a better understanding of the structure–function relationships in HemQ [1,11,12]. This includes Clds from perchlorate reducing bacteria (*Azospira oryzae* GR-1, *Ideonella dechloratans*, *Dechloromonas aromatica*, *Pseudomonas chloritidismutans*, *Magnetospirillum* sp.) [13–18], nitrite-oxidizing bacteria (‘*Candidatus* Nitrospira defluvii’, *Nitrobacter winogradskyi*) [12,19,20], from cyanobacteria (e.g. *Cyanothece* sp. PCC7425) [21] or pathogens like *Klebsiella pneumonia* MGH 78578 [22]. Mutational studies on Clds from ‘*Candidatus* Nitrospira defluvii’ (NdCld) [12,23] and from *D. aromatica* (DaCld) [24] revealed that the distal arginine–the only charged residue on the distal side of the haeme–is very important for chlorite degradation. It is conserved in Clds and DyPs, whereas in HemQs alanine, leucine, glutamine or serine can be found at this position (Figure 1A) [1,11,12]. HemQs do not show any chlorite degrading activity [4–6]. Interestingly, in mutants of NdCld and DaCld in which the distal Arg is exchanged by Gln [as in HemQs from the Firmicutes *Staphylococcus aureus* (SaHemQ) [4], *Bacillus subtilis* (BsHemQ) [3] and *Listeria monocytogenes* (LmHemQ) [5], the chlorite degrading activity is preserved to a certain extent but the mutants are more prone to irreversible inhibition. The latter phenomenon is closely related to the role of the distal Arg in retaining the transiently produced intermediate hypochlorite within the reaction sphere during enzyme turnover [25,26].

In both Clds and DyPs, the proximal haeme-coordinating histidine is hydrogen-bonded to a conserved negatively charged residue (glutamate in Clds, aspartate in DyPs). The H-bonding network in Clds is further extended by an H-bond between the glutamate and a conserved lysine that potentially forms another H-bond with a haeme propionate group (see Figure 1B for NwCld). Sequence alignments of Cld-like proteins and also structures of *Thermus thermophilus* (TtCld) [6] and LmHemQ [5] reveal that in HemQs either an alanine or a valine is found at the position of the acidic residue in Cld or DyP (Figure 1B). In contrast with Clds or DyPs, haeme binding in Cld-like proteins from TtCld, SaHemQ, BsHemQ and LmHemQ [3–6] is weak with reported *K*_D_-values being 30–40 μM in BsHemQ [3], 1 μM in SaHemQ [4] and 7–16 μM in LmHemQ [5]. For LmHemQ, it was shown that the protein is able to bind and release haeme reversibly, indicating a regulatory haeme sensing role during haeme biosynthesis in Gram-positive bacteria [5], as was also suggested by physiological studies of SaHemQ and BsHemQ [3,4]. Reversible haeme binding of the cofactor (substrate) is also a prerequisite for HemQs to act as coprohaeme decarboxylase (thereby binding coprohaeme and releasing protohaeme) [7].

Studies of proximal residue mutants of DaCld and NdCld revealed that manipulation of the rigid H-bonding network in Cld significantly weakens binding of haeme *b* and thereby destabilizes the protein [25–30]. In the present work, we studied the effect of disrupting the proximal H-bonding network by comparing wild-type NdCld with the proximal variants E210A and K141E. The first mutation mimics the HemQ-like proximal architecture. By using a broad set of spectroscopic techniques, including resonance Raman (RR) and electron paramagnetic resonance (EPR) spectroscopies, the impact of the proximal H-bonding network on haeme binding, overall thermal stability and enzymatic activity was evaluated in detail. The data demonstrate the role of the imidazolate character of the proximal histidine and the role of distal ligands in the modulation of binding and release of the prosthetic group. Our findings are discussed with respect to the known structure–function relationships of Clds and proposed biochemical function(s) of HemQ.

## MATERIALS AND METHODS

### Site-directed mutagenesis, overexpression and purification

Site-directed mutagenesis, production and purification of NdCld wild-type enzyme, E210A, K141E and R173Q/E210A variants were described in detail previously [23]. Briefly, a modified pET-21b(+) expression vector was used for the production of TEV-cleavable N-terminal Strep-II tagged fusion proteins. Heterologous expression was performed at 24°C for 4 h and 180 rpm shaking (after induction at an OD_600_ of approximately 0.6) in *E.coli* Tuner (DE3) cells (Merck/Novagen), 50 μg·ml^−1^ hemin was added prior to induction with 0.5 mM IPTG. Purification was performed by affinity chromatography using a StrepTrap column (GE Healthcare).

### Differential scanning calorimetry

Differential scanning calorimetric (DSC) measurements were performed using a VP-capillary DSC microcalorimeter from Microcal with a cell volume of 137 μl. The measurements were controlled by the VP-viewer program and the instrument was equipped with an autosampler for 96-well plates. Samples were analysed using a programmed heating scan rate of 60°C·h^−1^ over a temperature range from 20 to 120°C, cell pressure was approximately 60 psi (4.136 bar). DSC thermograms were corrected for buffer baseline and protein concentration. 5 μM of NdCld wild-type, E210A and K141E in 50 mM MES buffer, pH 5.5, was used for each measurement. The ligands were present in high excess (200 mM F^−^, 10 mM SCN^−^). For data analysis and conversion, the Microcal origin software was used. Heat capacity (*C*_p_) was expressed in kcal·mol^−1^·K^−1^. Data points were fitted to non-two state equilibrium-unfolding models by the Lavenberg/Marquardt (LM) non-linear least squares method.

### Electronic absorption spectroscopy

UV–visible electronic absorption spectra were recorded at 25°C between 250 and 700 nm on a Hitachi U-3900 UV–vis spectrophotometer. Temperature was controlled with a water bath connected to the cuvette-holder. The path length was 10 mm and the scan rate was 600 nm·min^−1^. Typically, 3 μM of the enzyme (NdCld wild-type, E210A, K141E) was measured in the absence and presence of excess fluoride (200 mM) and thiocyanate (10 mM) in 50 mM MES buffer, pH 5.5.

### Electron paramagnetic resonance spectroscopy

EPR spectroscopy was performed on a Bruker EMX continuous wave (cw) spectrometer, operating at X-band (9 GHz) frequencies. The instrument was equipped with a high sensitivity resonator and an Oxford Instruments ESR900 helium cryostat for low-temperature measurements. Spectra were recorded under non-saturating conditions using 2 mW microwave power, 100 kHz modulation frequency, 1 mT modulation amplitude and 20 ms conversion time, 20 ms time constant and 4096 points. Samples (100 μl of 30 μM) of recombinant NdCld wild-type, E210A and K141E were prepared in 50 mM MES buffer pH 5.5, transferred into Wilmad quartz tubes (3 mm inner diameter) and flash frozen in liquid nitrogen. In order to remove O_2_, the tubes were flushed with argon whereas the sample was kept frozen on dry ice. Measurements were performed at 10 K. The spectra were simulated with the Easyspin toolbox for Matlab [31] and consist of a weighted sum of simulations of the individual high-spin (HS) and low-spin (LS) species. The rhombicity was obtained from *g*^eff^_x_ and *g*^eff^_y_ and the relative intensities were calculated on the basis of the simulations, following the procedure of Aasa and Vanngard to account for the different integral intensity per unit spin of species that display different effective ***g*** values (as found in LS and HS centres) [32,33].

### Resonance Raman spectroscopy

RR spectra were measured with excitation at 413.1 nm (Kr+ laser, Coherent, Innova 300C) and 441.6 nm (HeCd laser, Kimmon IK4121R-G) using a triple spectrometer (consisting of two Acton Research SpectraPro 2300i working in the subtractive mode, and a SpectraPro 2500i in the final stage with a 3600 grooves·mm^−1^ grating), equipped with a liquid-nitrogen cooled CCD detector (Roper Scientific Princeton Instruments). RR spectra were calibrated with indene, *n*-pentane, dimethylsulfoxide, acetonitrile and carbon tetrachloride as standards to an accuracy of 1 cm^−1^ for intense isolated bands.

Absorption spectra (using a 5-mm NMR tube) were measured both prior to and after RR measurements to ensure that no degradation had taken place under the experimental conditions used. Electronic absorption spectra, measured with a double-beam spectrophotometer (Varian Cary 5), were recorded at a scan rate of 600 nm·min^−1^ scan rate. All RR measurements were repeated several times under the same conditions to ensure reproducibility. To improve the signal-to-noise ratio, a number of spectra were accumulated and summed only if no spectral differences were noted. All spectra were baseline corrected.

Samples of protein concentrations in the range 25–60 μM were used for the electronic absorption and RR samples in 0.1 M MES buffer, pH 5.5. The ferrous form was obtained by adding a small volume (2 μl) of a fresh sodium dithionite solution (10–20 mg·ml^−1^ Na_2_S_2_O_4_·2H_2_O) to 50 μl of a deoxygenated protein solution. The CO complexes were prepared by degassing the ferric protein solution by flushing firstly with nitrogen, then with CO or ^13^CO and reducing the haeme by addition of a 5% volume freshly prepared sodium dithionite (20 mg·ml^−1^) solution.

### Enzymatic activity

Cld-mediated chlorite reduction and concomitant oxygen generation was monitored by measuring the release of O_2_ using a Clark-type electrode (Oxygraph Plus; Hansatech Instruments). The electrode was inserted into a stirred water bath maintained at 30°C. The electrode was equilibrated to 100% O_2_ saturation by flushing with O_2_, further 0% saturation was achieved by bubbling with N_2_ until a plateau was reached. Reactions were carried out in O_2_-free 50 mM MES buffer, pH 5.5. The substrate (chlorite) concentration was 175 μM in the assay for each measurement added from a stock solution prepared in the same buffer. A high excess of ligands (200 mM F^−^, 10 mM SCN^−^) was present in the respective measurements. Reactions were started by the addition of the same amount (50 nM, final concentration) of NdCld wild-type, E210A and K141E. Molecular oxygen production rates (μM O_2_ s^−1^) were obtained from the initial linear time traces (initial velocities, *v*_0_) for direct comparison.

### Stopped-flow UV–visible spectroscopy

The experiments were carried out with a stopped-flow apparatus (model SX-18MV, Applied Photophysics) in the conventional mode. The optical quartz cell with a path length of 10 mm had a volume of 20 μl. The fastest mixing time was 1 ms. All measurements were performed at 25°C. Approximately 1 μM of wild-type, E210A and K141E, were mixed with 200 or 1000 μM chlorite in the absence and presence of a high excess of ligands (200 mM F^−^, 10 mM SCN^−^). Measurements were performed in 50 mM MES, pH 5.5, buffered solutions. The data were acquired for 20 s when no ligand or fluoride was present and for 60 s in the presence of 10 mM thiocyanate.

### Binding affinity of SCN^−^ and F^−^ to NdCld

SCN^−^ and F^−^ were titrated to NdCld wild-type for the determination of the dissociation constant (*K*_D_). The UV–visible electronic absorption spectrum of wild-type NdCld is altered upon binding of SCN^−^ or F^−^ if the respective ligand acts as a sixth ligand to haeme *b.* Binding of F^−^ was monitored by the change in absorbance at 612 nm [charge transfer band (CT1)] and binding of SCN^−^ was followed at 411 nm (Soret band). The measurements were performed using a Zeiss Specord S10 DiodeArray spectrophotometer equipped using a quartz cuvette with a path length of 10 mm under constant stirring at 25°C. Samples of 2 μM concentration in 50 mM MES buffer, pH 5.5, were used. Final ligand concentrations were 120 mM (F^−^) and 50 mM (SCN^−^).

### Haeme transfer

Release of haeme from wild-type NdCld and variants was tested by incubation of the respective proteins with horse heart apo-myoglobin (Sigma) [5,34]. Apo-myoglobin was prepared using a modified extraction method by Teale [35] as described previously [5]. Thirty micromolars of NdCld samples were incubated with 40 μM apo-myoglobin in 50 mM phosphate buffer, pH 7.0, for 1 h at room temperature. Size-exclusion chromatography was performed to separate NdCld and myoglobin. HPLC (Shimadzu prominence LC20) was equipped with a refractive index detector (RID-10A, Shimadzu), a diode array detector (SPD-M20A, Shimadzu) and a MALLS-detector (Multi-Angle-Laser-Light-Scattering; WYATT Heleos Dawn8+ plus QELS; software ASTRA 6). The column (Superdex 200 10/300 GL, GE Healthcare) with particle size of 13 μm was equilibrated with the running buffer (Dulbecco PBS, 200 mM NaCl). The experiments were performed at a flow rate of 0.75 ml·min^−1^, 80 μl of the protein solution (containing 30 μM NdCld and 40 μM apo-myoglobin) were injected. Untreated NdCld samples (wild-type, K141E, E210A, R173Q/E210A), holo- and apo-myoglobin as well as Bio-Rad Gel Filtration Standard (#151-1901) containing also holo-myoglobin from horse heart, were injected as references. Haeme transfer was observed by extracting elution profiles at 280 and 409 nm from the diode array detector.

## RESULTS

### Distal ligands significantly increase the thermal stability of wild-type NdCld and proximal mutants

**Figure 2:**
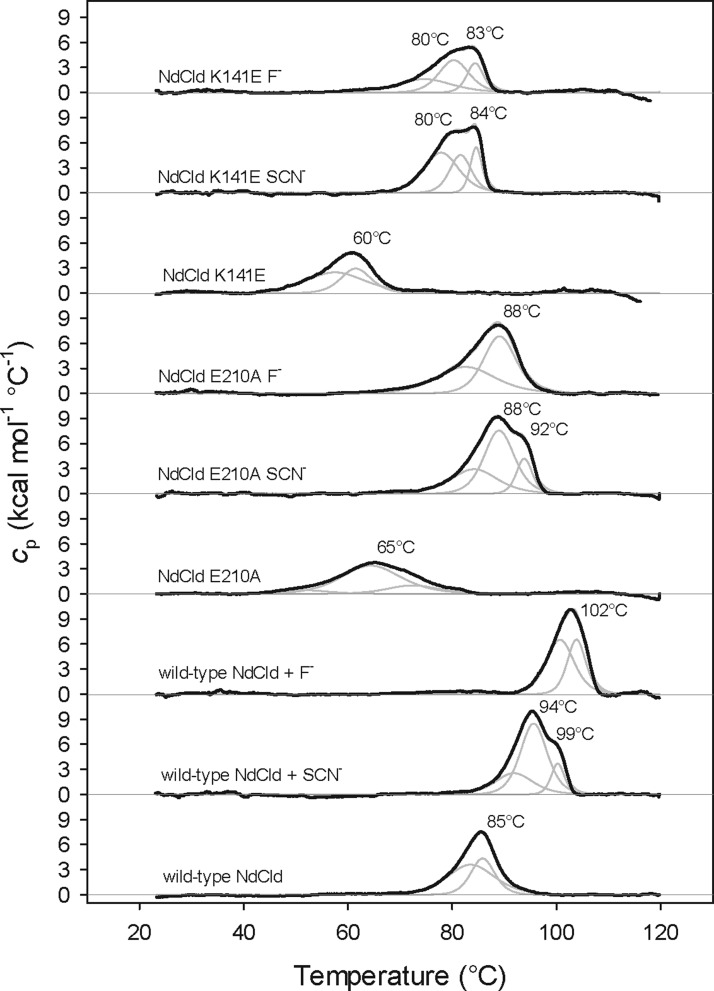
DSC of wild-type NdCld and the variants E210A and K141E Ligand free thermograms are compared with those in the presence of 10 mM thiocyanate (SCN^−^) or 200 mM fluoride (F^−^). Thermal transitions were fitted to non-two state equilibrium-unfolding models by the Lavenberg/Marquardt (LM) non-linear least squares method and fits are depicted as grey lines.

In order to test the impact of the introduced proximal mutations, differential scanning calorimetry (DSC) was performed. Wild-type pentameric NdCld is known to be a highly stable enzyme with a melting temperature of around 90°C in phosphate buffered solutions [36]. In 50 mM MES buffer, pH 5.5, the *T*_m_-value is slightly lower (85°C). The variants E210A and K141E are significantly less thermostable showing broad transitions with *T*_m_-values of 65 and 60°C respectively (Figure 2).

Importantly, the addition of SCN^−^ and F^−^ has a significant stabilizing effect on both the wild-type and mutant proteins. The thermogram of wild-type NdCld in the presence of SCN^−^ exhibits a peak maximum (*T*_m_-value) at 94°C with a shoulder at 99°C, whereas in the presence of F^−^, the enzyme has a melting temperature of even 102°C. The thermogram of NdCld E210A in the presence of SCN^−^ showed a non-two-state transition with peak maxima at 88 and 92°C, whereas in the presence of F^−^, the peak is sharper (*T*_m_ at 88°C). Complex formation of the variant K141E by addition of thiocyanate and fluoride gives broad peaks with maxima at 80°C as well as 83 or 84°C respectively (Figure 2). All fitted peaks in the thermograms exhibit cooperative unfolding patterns (Δ*H*_v_/Δ*H* > 1). On average the first peak has a Δ*H*_v_/Δ*H* ratio of 2.4 (corresponding to the unfolding of two entities; most probably of two pentamers) and the second peak of 5.4 (corresponding to the unfolding of pentamers). These data fit well to the HPLC profiles (see below Figure 9). If a third peak occurs (highest *T*_m_), which is the case upon addition of thiocyanate for all proteins and for fluoride in K141E, the Δ*H*_v_/Δ*H* ratio is significantly higher (19.8). This indicates that a certain amount highly stable arrangements of higher oligomeric states are formed.

### Effect of disruption of proximal H-bonding on the spin states of the ferric proteins

**Figure 3:**
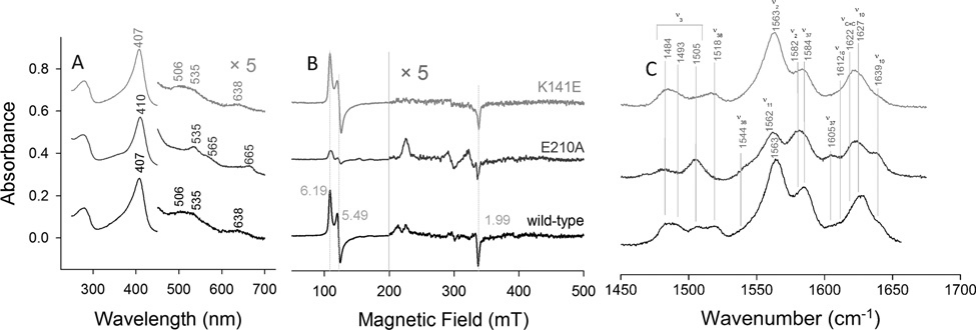
Spectroscopic signatures of ferric wild-type NdCld and the variants E210A and K141E (**A**) UV–visible absorption, (**B**) EPR and (**C**) RR spectra (*λ*_excitation_=413.1 nm, laser power at the sample 5 mW, average of 2 spectra with 300 s integration time). Conditions: 50 mM MES, pH 5.5.

The UV–vis and RR data of wild-type NdCld indicate that it is a mixture of 5-coordinate (5c), 6-coordinate (6c) HS and 6c LS species at pH 5.5. This is evident from the Soret maximum at 407 nm, Q-bands at 506 and 535 nm, CT1 at 638 nm (Figure 3A) and high frequency RR spectra that show the presence of 6cHS, 5cHS and 6cLS states (ν_3_ modes at 1484, 1493 and 1505 cm^−1^, respectively) (Figure 3C). The complete assignment of the RR bands is given in Table 1. In agreement with the UV–vis and RR data, the EPR spectrum shows a mixture of a predominantly rhombic HS signal, a minor axial HS signal and two LS species (Figure 3B and Supplementary Table S1). The ***g*** values and relative populations of the four species obtained by simulation of the EPR spectrum are presented in Table S1. The observed spin states in wild-type NdCld are in agreement with previously published UV–vis and low-temperature (10 K) EPR spectra [23,25,37]. It is noted that the ***g*** values of both LS species and particularly the HS states are slightly different from those previously reported for NdCld [37], possibly due to the use of phosphate buffer that may lead to variations in buffer pH at low temperature [38]. As has been observed for other Clds, EPR signals very similar to the LS species of NdCld, particularly LS2, reported herein have been suggested to result from binding of imidazoles or histidine residues [39,40]. The thorough EPR analysis to determine the origin of such signals in Cld from *Magnetospirillum* sp. by van Doorslaer excluded histidine ligation but could not offer a conclusive assignment of the LS species [40]. It was suggested that a buffer molecule may lead to the formation of the LS species, possibly stabilized by H-bonding with the distal Arg residue, though this proposal could not be confirmed. In the present case, histidine ligation can also be excluded as no His residue is present on the distal side of the haeme pocket and the use of a StrepII-tag ensured that none of the imidazole used in the purification process remained in the purified protein.

**Table 1:** Vibrational assignment of the RR bands* observed for ferric NdCld at pH 5.5 *Frequency in cm^−1^. ^†^These bands overlap.

Mode	Symmetry	6cHS	5cHS	6cLS
*ν*_4_	A_1g_		1372	
*ν*_3_	A_1g_	1484	1492	1505
*ν*_38_	E_u_	1517		1544
*ν*_11_	B_1g_			1562
*ν*_2_	A_1g_	1563		1582
*ν*_37_	E_u_	1584		1605
*ν*_10_	B_1g_	1612	1627^†^	1639
*ν*_(c=c)_		1622^†^		

Disruption of the H-bond between the proximal His^160^ and Glu^210^ residues (variant E210A) increases significantly the amount of 6cLS haeme. This is reflected by i) red-shifted Soret (410 nm) and Q-bands (535 and 565 nm) in the UV–visible spectrum (the wavelengths of the distinct Q-bands suggest the presence of a bis-N species) [41], ii) an increase in intensity of the LS species with the concomitant decrease in HS signals in the EPR spectrum, iii) a significant decrease in intensity of the 5cHS and 6cHS bands in the RR spectrum (Figure 3). Contrary to wild-type NdCld, only one LS EPR species (LS1) is observed in the E210A variant. Conversely, compared with the wild-type protein, the UV–vis, RR and EPR data indicate that the 6cLS is strongly reduced by exchange of the proximal lysine with a glutamate (K141E) (Figure 3 and Supplementary Figure S1, Supplementary Table S1). The weak band at approximately 665 nm, observed in all the UV–vis spectra of the E210A mutant, is assigned to an impurity probably due to a haeme *d* species [42].

### Spectral studies on the fluoride and thiocyanate complexes

As outlined above, addition of both fluoride and thiocyanate significantly increases the thermal stability of wild-type NdCld and the variants E210A and K141E. Thiocyanate and fluoride act as weak ligands. The respective *K*_D_-values of the wild-type protein are 150±40 μM (SCN^−^) and 5.9±0.4 mM (F^−^) at pH 5.5 (Supplementary Figure S2).

**Figure 4:**
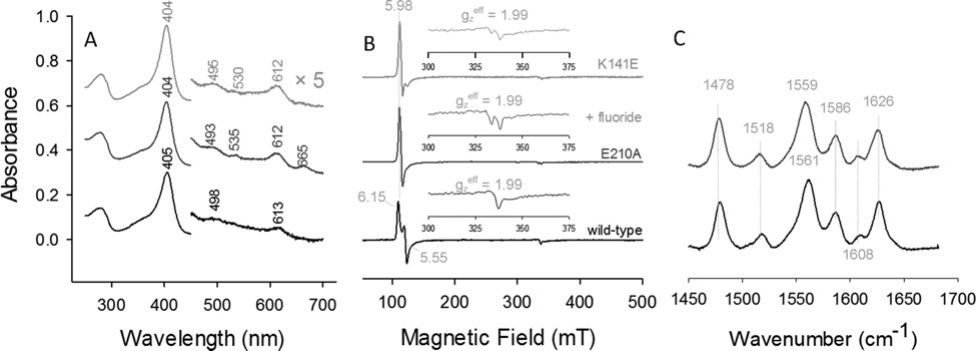
UV–vis, EPR and RR spectra of ferric wild-type NdCld and the variants E210A and K141E bound to fluoride (**A**) UV–visible absorption spectra. (**B**) Low-temperature EPR spectra. (**C**) RR spectra (*λ*_excitation_=413.1 nm, laser power at the sample 1.5 mW, average of 2 spectra with 40 s integration time). Conditions: 50 mM MES, pH 5.5.

Fluoride acts as a HS ligand and the UV–vis spectra of wild-type NdCld and variants are consistent with pure 6cHS enzymes characterized by Soret bands at 404 nm (variants) or 405 nm (wild-type NdCld) and a CT1 band at 612–613 nm (Figure 4A). The CT1 band of haeme protein fluoride complexes ranges between 602 and 619 nm. A red-shift of the CT1 band indicates the presence of H-bonding interactions between fluoride and distal cavity residues [43]. In NdCld, the distal residue that H-bonds with the fluoride ligand is most probably the catalytically important distal arginine (Arg^173^), since the variants (R173A, R173K, R173Q, R173E) [12,23,24] are unable to bind fluoride (data not shown). The RR spectra of the wild-type and E210A fluoride derivatives also show that fluoride binding leads to the formation of a pure 6cHS state (*ν*_3_ 1478, *ν*_2_ 1561, *ν*_10_ 1608 cm^−1^; Figure 4C).

Surprisingly, the EPR spectrum of the fluoride complex of wild-type NdCld exhibits i) an increased amount of 5cHS, ii) an invariant level of 6cHS, iii) reduced levels of both LS species, iv) absence of the diagnostic doublet at ***g***=2, due to superhyperfine coupling with the ^19^F nucleus, typical of fluoride bound haeme derivatives [32] (Figure 4B; Table S1). It is noted that upon thawing the sample after the EPR measurement, the UV–vis spectrum was identical with that of Figure 4(A). Hence, at the temperature of the EPR measurement (10 K), there must be a structural rearrangement of the distal cavity that impedes fluoride binding. Significant changes in EPR spectral features were observed upon addition of fluoride to the E210A mutant. The LS signal is considerably reduced and a predominant axial 6cHS signal is observed. Additionally, band splitting in the ***g***=2 region reflects super-hyperfine coupling with the bound fluoride nucleus (Figure 4B). In the fluoride complex of the K141E variant, the axial 6cHS signal is the dominant species, although a certain amount of rhombic 5cHS signal is still present. Again, the EPR signal in the ***g***=2 region clearly shows coupling with the fluoride nucleus (Figure 4B). The hyperfine coupling with the ^19^F nucleus determined by spectral simulation for E210A and K141E was 48 and 44 G, respectively. As noted above for the wild-type protein, the apparent incomplete consistency between the EPR data, which indicate that the 6cHS state is not pure (Table S1), and the room temperature UV–vis and RR data (see Figures 4A and 4C), which suggest complete binding of the fluoride ligand to the variant proteins, probably results from temperature induced structural changes. All the experimental spectra superimposed on their simulated spectra are shown in Supplementary Figure S1 and the EPR parameters derived from the simulations are listed in Table S1.

**Figure 5:**
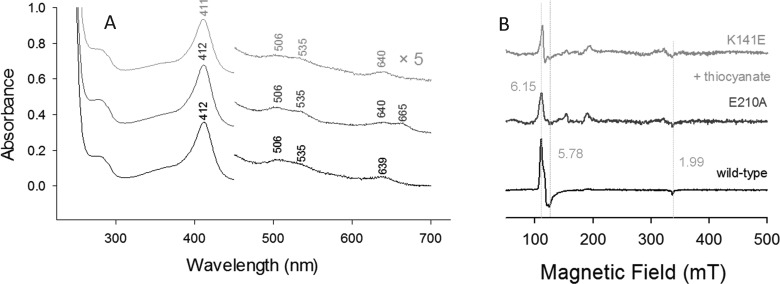
UV–vis and EPR spectra of ferric wild-type NdCld and the variants E210A and K141E bound to thiocyanate (**A**) UV–visible absorption spectra. (**B**) Low-temperature EPR spectra. Conditions: 50 mM MES, pH 5.5. The EPR spectra in the region 200–500 mT have been expanded 5-fold.

The UV–visible electronic absorption spectra of the thiocyanate complex of wild-type NdCld and the variants exhibit red-shifted Soret maxima (411–412 nm), Q-bands at 506 and 535 nm and a CT1 band at 638 nm. Whereas the strong CT1 band indicates the presence of 6cHS ferric haeme, the red-shifted Soret band is indicative of 6cLS ferric haeme. The EPR spectra support the presence of a mixed spin population (Figure 5B, Supplementary Table S1). The amount of LS formed by thiocyanate binding to the haeme is low in wild-type NdCld (36%) and high in both the E210A (84%) and K141E (87%) variants.

### Impact of disruption of the proximal H-bonding network on the bond strength between the proximal histidine and the haeme

**Figure 6:**
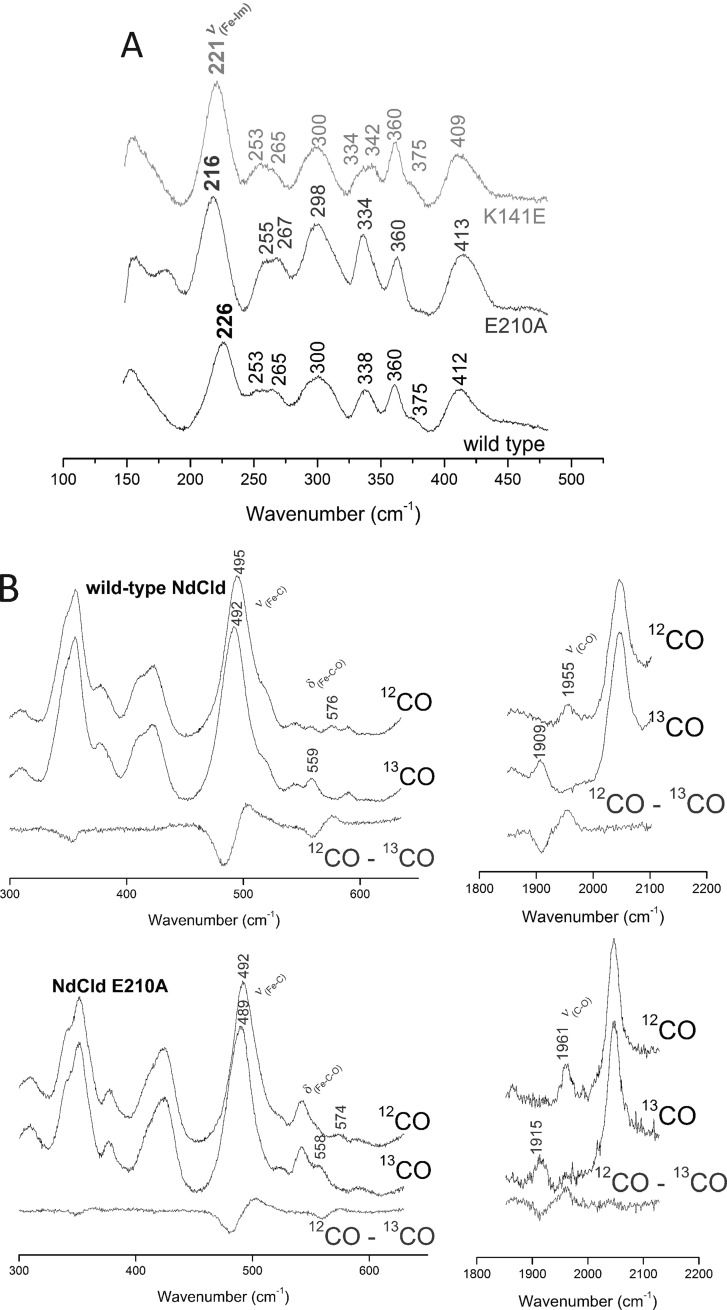
Resonance Raman spectra of ferrous NdCld and variants (**A**) Low frequency resonance Raman spectra of ferrous wild-type NdCld and the variants E210A and K141E. Excitation wavelength: 441.6 nm, laser power at the sample 11 mW, average of three spectra (wild-type) and two spectra (variants) with 600 s integration time. (**B**) Low and high frequency resonance Raman spectra of the wild-type NdCld and the variant E210A complexes with CO and ^13^CO. Difference spectra are depicted in red. Excitation wavelength: 413.1 nm, laser power at the sample 1 mW, spectra in the low frequency region average of 3 spectra with 600 s integration time, in the high frequency region average of 6 spectra (wild-type, CO), and 12 spectra (wild-type, ^13^CO) with 240 s integration time, 6 spectra (NdCld E210A, CO) and 5 spectra (NdCld E210A, ^13^CO) with 600 s integration time.

Upon reduction, wild-type NdCld and mutated proteins at pH 5.5 display electronic absorption and high frequency RR spectra that are typical of a pure 5cHS haeme (data not shown). Hence, it is clear that the ligand which binds the haeme iron in the ferric state, leading to the LS species, is unable to coordinate the haeme in the reduced wild-type and variant proteins. The low frequency RR spectra of ferrous 5cHS haeme proteins are of particular interest due to the presence of a strong band in the range 200–250 cm^−1^ due to the iron–imidazole stretching mode, *ν*(Fe–Im) (representing the bond strength between the haeme iron and the proximal histidine). Its frequency is an optimum probe of the proximal cavity structure as it is very sensitive to the protein matrix. In fact, it is strongly affected by the H-bonds between the N_δ  _atom of the proximal His and nearby residues [44,45]. The intense band at 226 cm^−1^ observed for ferrous wild-type NdCld at pH 5.5 (Figure 6A), which strongly decreased in the RR spectrum taken with 413.1 nm excitation (i.e. not in resonance with the Soret band; data not shown) is assigned to the *ν*(Fe–Im) mode. This frequency is slightly higher than that observed in myoglobins (220 cm^−1^) [46], where the proximal histidine is H-bonded to a weak H-bond acceptor. The frequency is also higher than in Cld from *D. aromatica* (DaCld) (222 cm^−1^) [47], but lower than in Cld from *K. pneumonia* MGH 78578 (KpCld) (229 cm^−1^) [22].

Since the presence of H-bonds with a nearby residue increases the *ν*(Fe–Im) frequency, the high 226 cm^−1^ frequency is due to the H-bond between the proximal histidine and the neighbouring glutamate which imparts imidazolate character. Accordingly, the loss of this H-bond in the variant E210A results in the lowering of the *ν*(Fe–Im) mode frequency by 10 cm^−1^ (216 cm^−1^, Figure 6A), suggesting that His^160^ loses its imidazolate character. In the variant K141E, the corresponding band is found at 221 cm^−1^, which underlines the role of Lys^141^ in fine tuning and stabilization of the proximal H-bonding network in NdCld. The hierarchy of bond strength between the haeme iron and His^160^ is wild-type NdCld > K141E > E210A.

### CO binding to wild-type NdCld and the variants E210A and K141E

The addition of CO to the ferrous forms of the wild-type protein and its variants gives rise to electronic absorption spectra typical of 6cLS forms, with CO bound as the sixth ligand of the iron atom (data not shown). This confirms the observation that the substantial proportion of LS species present in the ferric forms is absent from the reduced proteins. The RR spectra in the low frequency region of wild-type ferrous NdCld complexed with CO (Figure 6B) shows two isotope-sensitive bands at 495 and 576 cm^−1^, which shift to 492 and 559 cm^−1^ upon ^13^C-labelling of carbon monoxide, assigned to the *ν*(Fe–C) stretching and *δ*(Fe–C–O) bending modes, respectively. Accordingly, a corresponding *ν*(CO) stretching mode is observed at 1955 cm^−1^, which shifts to 1909 cm^−1^ in the ^13^CO complex (Figure 6B). These frequencies do not vary in the pH range 5.5–9.8. The observed *ν*(Fe–C)/*ν*(CO) frequencies are typical of haeme-CO adducts that have little interaction with the distal protein matrix. In horseradish peroxidase C (HRP-C), where a distal histidine and a distal arginine interact with the bound CO, the *ν*(Fe–C) and *ν*(CO) stretching mode frequencies are found at 539 and 1906 cm^−1^ respectively [48]. These findings are in agreement with recent computational studies demonstrating that the mobile distal side Arg^173^ points away from the haeme in ferrous NdCld [26].

Although the frequencies of the isotope-sensitive bands of the variant K141E are identical with those of the wild-type enzyme (data not shown), those of E210A are slightly different [*ν*(Fe–C) at 492 cm^−1^, *δ*(Fe–C–O) at 574 cm^−1^ and *ν*(CO) at 1961 cm^−1^] (Figure 6B). The slight down-shift of the *ν*(Fe–C) stretching frequency together with the up-shift of the *ν*(CO) stretching mode compared with the wild-type protein indicate a slight decrease in the interaction between the bound CO and the distal haeme environment in E210A compared with the wild-type protein and K141E.

### Impact of H-bond disruption on chlorite degradation and enzyme inhibition

Recently, steady-state kinetic parameters for the degradation of chlorite by wild-type NdCld and various mutants (including E210A and K141E) were reported [12,23]. At pH 7.0, the catalytic efficiencies (*k*_cat_/*K*_M_) for wild-type NdCld and the variants E210A and K141E are 6.2×10^5^, 1.2×10^5^ and 4.6×10^5^ M^−1^·s^−1^ respectively.

Wild-type NdCld exhibits the highest catalytic efficiency at pH 5.5 (*k*_cat_/*K*_M_=1.5×10^6^ M^−1^·s^−1^). It has been demonstrated that (i) during turnover the enzyme is irreversibly inhibited with time due to partial release of the highly reactive intermediate hypochlorite from the reaction sphere [25] and that (ii) this inhibition reaction becomes more pronounced with increasing pH. Herein, we have analysed the effect of disruption of the proximal H-bonding network on these reactions. The pH optimum for both E210A and K141E is wild-type-like. To analyse the effect of mutation on inhibition, the enzymatic activity of 50 nM wild-type protein and variants was probed under identical protein to chlorite ratios at pH 5.5. Furthermore, the impact of 200 mM fluoride and 10 mM thiocyanate on chlorite degradation was tested. Reaction rates were calculated only from the initial reaction phase in order to minimize the influence of inhibition.

**Figure 7:**
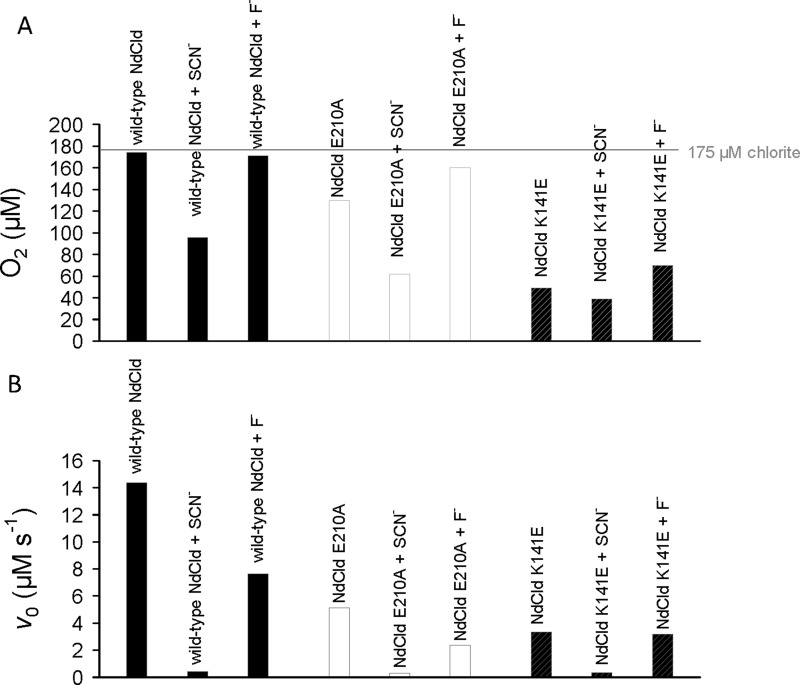
Steady-state kinetics of chlorite degradation by wild-type NdCld (black) and the variants E210A (white) and K141E (striped) (**A**) Total concentration of released dioxygen. (**B**) Initial velocities of O_2_ generation. Measurements were performed in 50 mM MES buffer, pH 5.5 using 50 nM of enzyme and 175 μM chlorite as substrate.

Wild-type NdCld is able to entirely convert 175 μM chlorite with an initial velocity (*v*_0_) of 14.4 μM·s^−1^ (Figure 7). In the presence of fluoride, *v*_0_ is reduced to 53% (7.6 μM·s^−1^) but chlorite is still fully degraded. By contrast, in the presence of thiocyanate the reaction is much slower (<0.5 μM·s^−1^) and only 55% of the substrate is converted before the enzyme is irreversibly inhibited. The variant E210A is able to convert 75% of chlorite at a *v*_0_ of 5.1 μM·s^−1^ prior to self-inactivation. Initial velocities in the presence of thiocyanate were <0.5 μM·s^−1^ and only 35% of the substrate was degraded. Interestingly, fluoride protects this mutant from early self-inactivation with 95% of chlorite being converted to chloride and dioxygen at an initial velocity of 2.3 μM·s^−1^ (Figure 7). The variant K141E degrades 28% of chlorite in the absence of a ligand, 22% in the presence of thiocyanate (<0.5 μM·s^−1^) and 40% in the presence of fluoride (3.2 μM·s^−1^).

**Figure 8:**
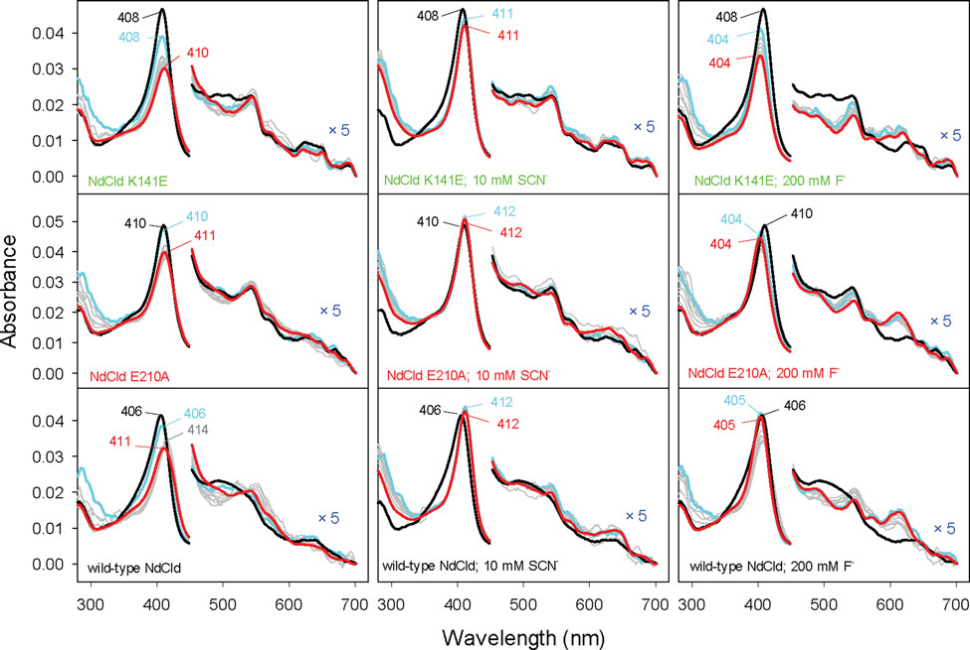
Transient-state kinetics of chlorite degradation by wild-type NdCld and the variants E210A and K141E 500 nM of enzyme were mixed with 200 μM chlorite (final concentration) in the absence or presence of 10 mM thiocyanate or 200 mM fluoride. Spectra from the following time points are shown: 0 (black), 1 (cyan), 51, 101, 221, 446, 866, 1646, 3099, 5803, 10838 (grey) and 20 s (red).

These reactions were also followed by stopped-flow spectroscopy in order to analyse interconversion and the electronic state of the relevant redox intermediates (Figure 8, Supplementary Figure S3). In Figure 8, the ferric resting enzyme is depicted in black, the first spectrum monitored 1 ms after mixing with chlorite (final concentrations either 200 or 1000 μM) is shown in cyan and the final spectrum, monitored after 20 or 60 s, in red. During very rapid degradation of 200 μM chlorite (*ε*_260nm_=155 M^−1^·cm^−1^) [49] at *k*_obs_ of 8.7 s^−1^, wild-type NdCld loses Soret absorbance. This suggests haeme bleaching as reported previously (Figure 8) [25]. During turnover, the dominating redox intermediate has a Soret maximum at 414 nm (with Q-bands typical for LS haeme including loss of the CT1 band). Haeme bleaching is more pronounced at higher chlorite concentrations (Figure S3). In the presence of 10 mM SCN^−^, chlorite degradation was significantly slower and haeme bleaching was observed only at high chlorite concentrations (compare Figure 8, Supplementary Figure S3C). Upon addition of 200 mM fluoride, wild-type NdCld formed a 6cHS haeme complex immediately (1 ms) after mixing and was able to convert 200 μM chlorite entirely without haeme bleaching at *k*_obs_ of 3.6 s^−1^ (Figure 8). At the end of the reaction, the spectrum of the enzyme–fluoride complex was fully restored. During reaction, the dominating intermediate exhibits reduced Soret and CT1 band intensity but increased absorbance in the Q-band region at 535 nm (grey bands). At 1000 μM chlorite, haeme bleaching was observed, but is less pronounced compared with that in the absence of fluoride (Supplementary Figure S3).

Spectral transitions of the variants E210A and K141E in the absence and presence of thiocyanate or fluoride during the reaction with 200 or 1000 μM chlorite are qualitatively comparable to those observed for the wild-type enzyme. Ferric E210A is in a predominantly LS state as described above (Figure 8) and degrades chlorite slowly. Already at 200 μM substrate, haeme degradation is observed. The mutant K141E shows the least stability during enzymatic turnover, indicated by rapid haeme bleaching and self-inactivation (Figure 8), which is in agreement with the steady-state kinetics described above (Figure 7). For both variants, the presence of ligands has a protective effect on haeme degradation.

### Impact of proximal mutations on the release of the prosthetic group

To enable catalysis, the prosthetic group has to be incorporated into the active site in a stable way in functional Clds, whereas in HemQ the postulated functional role includes binding of a precursor molecule and release of the product haeme *b*. Sequence alignment together with structural data suggest that the proximal haeme architecture mainly contributes to the dissociation constant of the respective protein–haeme *b* complex and this hypothesis is strongly supported by the data presented above. Recently, it was demonstrated that the prosthetic group in HemQ from *L. monocytogenes* can easily be extracted by addition of apo-myoglobin [5]. Herein, we have performed similar experiments in order to analyse the effect(s) of manipulation of the proximal H-bonding network of NdCld on haeme binding. Besides the single variants E210A and K141E, the distal/proximal double mutant R173Q/E210A was investigated, since it resembles the haeme environment of LmHemQ.

**Figure 9:**
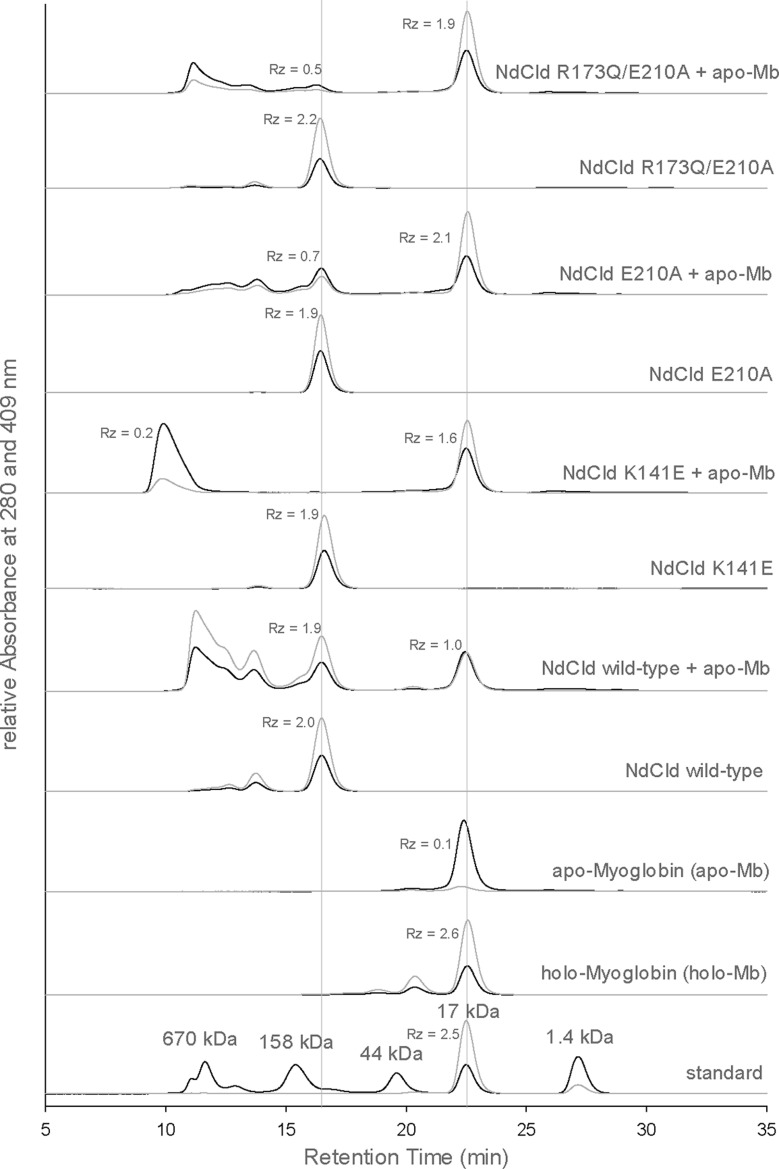
Haeme transfer from wild-type NdCld and variants to apo-myoglobin HPLC chromatograms (from bottom to top) of standard proteins, holo-myoglobin, apo-myoglobin and NdCld samples (wild-type, K141E, E210A, R173Q/E210A) in the absence and presence of apo-myoglobin. Elution profiles followed at 280 and 409 nm (Soret absorbance) are depicted in black and grey respectively.

Whereas no spectral shifts in the UV–vis spectrum are observed upon mixing of wild-type NdCld with apo-myoglobin, a clear spectral shift from 407 to 409 nm (i.e. the Soret maximum of holo-myoglobin) is observed when any of the three mutant proteins is incubated with apo-myoglobin (not shown). These findings clearly suggest haeme transfer from the Cld mutants to apo-myoglobin. To analyse this in more detail, an HPLC-based assay was developed (Figure 9). Cld (30 μM wild-type or mutants) was incubated with a slight molar excess of apo-myoglobin (40 μM) for 1 h before separating the proteins by size exclusion chromatography. Because of the considerable differences in molar masses, the elution peaks of pentameric NdCld and (monomeric) myoglobin are well separated. Moreover, haeme occupancy has only a small impact on the elution time of the respective proteins (Figure 9). Interestingly, addition of apo-myoglobin to wild-type NdCld yields myoglobin with 38% haeme occupancy (*R*_z_ value of 1.0) (Figure 9). Upon incubation of the two proteins, some aggregation of NdCld is observed which might be caused by released and unspecifically bound haeme moieties. In accordance with the spectroscopic studies, haeme transfer from the three mutant proteins to apo-myoglobin was much more pronounced following the hierarchy wild-type NdCld < K141E < R173Q/E210A < E210A.

## DISCUSSION

Incorporation and stabilization of the haeme co-factor can be achieved and controlled in various ways. Human peroxidases, like lactoperoxidase, eosinophil peroxidase and thyroid peroxidase autocatalytically form covalent haeme to protein ester linkages [50], whereas in myeloperoxidase an additional sulfonium linkage between a haeme vinyl group and a methionine is found [51]. Recently, it was shown that also some bacterial peroxidases establish covalent haeme to protein ester bonds [52,53]. However, in most haeme proteins the Fe-proximal His represents the only covalent bond between the haeme chromophore and the protein, and stable incorporation of the prosthetic group occurs via several non-covalent interactions. These include H-bonding networks at the proximal side [54] as shown for haeme *b*-containing Clds [12,20,30,55].

The most obvious difference between Clds and Cld-like proteins concerns amino acids in the immediate vicinity of the proximal ligand histidine (H160), which is glutamate (E210) in functional Clds and either alanine or valine in Cld-like proteins (Figure 1) [5,6]. Close to the proximal His in both protein families is a fully conserved lysine, which in the case of Clds allows the establishment of a continuous H-bonding network from the proximal histidine to the propionate substituent of the haeme. Upon designing the NdCld variant E210A, we aimed at mimicking the haeme cavity architecture of Cld-like proteins. An evaluation of the impact of the disturbance triggered by exchange of K141 with a negatively charged residue can contribute to our understanding of the structure–function relationships of the whole superfamily.

The importance of both E210 and K141 for maintaining an overall stable fold of NdCld is demonstrated convincingly by the drastically reduced thermal stabilities of the variants E210A and K141E. Upon exchange of E210 by alanine, the proximal histidine loses its imidazolate character. This significantly weakens the bond strength between the haeme iron and H160 (located in the α3'-helix) as shown by RR spectroscopy of the ferrous proteins. This effect is striking in E210A, but is also observed in K141E (Figure 6A). For another functional chlorite dismutase, namely DaCld, the observed *ν*(Fe–Im) stretching band (222 cm^−1^) suggests weaker binding between the haeme iron and its proximal ligand compared with NdCld [47]. Even though no direct data concerning the stability of DaCld are available, indirect methods and observations support a lower conformational and thermal stability of DaCld compared with NdCld [24,27,30]. Retrospectively, this correlates with the observed *ν*(Fe–Im) stretching frequency of both functional Clds.

Interestingly, as previously found for haeme containing peroxidases [56–58], proximal mutation affects the distal side environment. In the ferric proximal E210A variant, the LS species increase significantly compared with the wild-type protein, becoming the predominant state. Although the spectral features of the LS state are reminiscent of an N-ligated haeme iron (Figure 3; Supplementary Table S1), its origin is unclear, as previously reported for Cld from *Magnetospirillum* sp.; the LS ligand possibly being stabilized by H-bonding with the flexible distal arginine [40]. In fact, it is known that the distal Arg is able to change between two conformations, either pointing towards or away from the haeme iron [24–26]. This hypothesis is consistent with the fact that in wild-type NdCld a substantial proportion of LS haeme iron is present, whereas exchange of the distal arginine by alanine gives HS haeme only, as clearly indicated by the absence of the bands at 535 and 570 nm in the mutant (Supplementary Figure S4). In the E210A variant the architecture of the haeme cavity is modified; in particular, the distance of the haeme to the β-sheet (β2') on the distal side is reduced [12]. As a consequence, the distal arginine is in closer proximity to the haeme, thereby causing an increased amount of LS state. Insertion of a negatively charged residue in the variant K141E does not alter dramatically the spectral features of the ferric state, which are similar to the wild-type protein at room temperature; however, the low temperature EPR spectrum displays a significantly reduced proportion of LS species suggesting that the distal arginine is more distant from the haeme (Figure 3; Supplementary Table S1).

It is interesting to note that fluoride bound at the distal haeme side has a multiple impact on the behaviour of both wild-type and mutant Clds. It significantly increases the thermal stability and forms pure 6cHS species at room temperature eliminating the LS species. Fluoride is an excellent probe to test H-bonding interactions in the distal cavity of haeme proteins since it acts as a weak HS ligand stabilized by H-bonding with distal residues [43,59]. In the case of NdCld, the distal arginine is the only potential distal H-bonding partner. Upon fluoride binding, in both wild-type NdCld and the variant E210A, the LS UV–vis and RR spectral markers disappear (Figure 4). The EPR analysis (Supplementary Figure S1, Supplementary Table S1) is complicated by the structural variations that are apparently induced in the haeme cavity at low temperature that lead to reduced (in the mutants), or absent (in the wild-type protein), fluoride binding.

Changes in the distal site architecture upon disruption of proximal H-bonds are also reflected by carbon monoxide binding studies that showed a slight decrease in the interaction between the bound CO and the distal haeme environment in E210A compared with wild-type NdCld (Figure 6B). Generally, in ferrous NdCld the distal arginine points away from the haeme, as shown in MD simulation studies [26], and thus the interactions of CO with the distal cavity are weak.

Furthermore, manipulation of proximal residues strongly influences the kinetics of substrate conversion that occurs at the distal side, as reflected by the diminished catalytic efficiencies (*k*_cat_/*K*_m_) of the variants E210A and K141E compared with wild-type NdCld. It should be noted that calculation of the *K*_m_ and *k*_cat_ values is influenced by the respective enzyme to chlorite ratios due to irreversible self-inactivation [25]. Hence, the kinetic parameters must be determined from the initial reaction phase only. Interestingly, fluoride not only stabilizes the overall fold but also protects NdCld from early self-inactivation by slowing down initial rates of chlorite degradation. A possible explanation may be that the small halide regulates the rate of chlorite binding to ferric Cld and, consequently, also oxidation of the ferric protein to Compound I. In the course of this reaction, hypochlorite is transiently produced [25,26]. Typically, this half reaction is significantly faster than the following recombination reaction between the oxoferryl species of Compound I and hypochlorite (leading to the formation of chloride and dioxygen) [25,26], since the dominating redox intermediate during turnover is a LS species. However, the system is partially leaky and some hypochlorite is released and causes oxidative damage to both the protein and the prosthetic group [25,26]. By decreasing the rate of hypochlorite formation, fluoride indirectly diminishes its release from the reaction sphere. Both variants E210A and K141E are more prone to self-inactivation during chlorite degradation than the wild-type protein. Again, this reflects the importance of an intact distal side architecture for optimal coordination of transiently formed hypochlorite. Also in case of the mutants, fluoride provides protection from self-inactivation, at least to some extent. The protective effect was smallest for the variant K141E, which underlines the fragility of its proximal architecture induced by the change of a basic to an acidic residue. Qualitatively, the effect of thiocyanate is similar to that of fluoride, but due to its higher affinity it blocks chlorite binding more efficiently and decelerates the reaction drastically.

In summary, the present data clearly demonstrate that exchange of the (Cld-typical) proximal glutamate by alanine (as found in Cld-like proteins) destabilizes the overall protein fold and modifies the haeme cavity architecture. The mutation weakens haeme binding to the protein thereby allowing the transfer of haeme *b* to an acceptor protein like apo-myoglobin. We also demonstrated that the availability of a HS ligand can compensate this instability and render the enzyme properties wild-type-like. Since the overall subunit fold of proteins within the peroxidase-Cld family (structural CDE superfamily) is highly conserved [1,2,60] and Cld-like proteins (HemQs) and Clds share similar active site architecture, our study has provided a better insight into the biochemistry of Cld-like proteins. The most striking difference between Cld-like proteins, HemQs, and functional Clds is the catalytic distal arginine which is fully conserved only in the latter. In its absence, chlorite degradation still occurs but at a very low rate (as long as the H-bonding network H160-E210-K141 is intact) [12,23,24]. Interestingly, no chlorite degrading activity was detected in Cld-like proteins of *T. thermophilus* and HemQs of *S. aureus* and *L. monocytogenes* (LmHemQ) [4–6]. These proteins lack the distal arginine, have a neutral proximal histidine and thus bind the haeme group very weakly compared with functional Clds. In fact, in a previous study, we showed that the NdCld double mutant R173Q/E210A, which mimics the haeme environment of LmHemQ, almost completely lost the ability to degrade chlorite or bind the LS ligand cyanide [23]. Concomitantly, this mutant easily donates its prosthetic group to an acceptor protein with significantly higher affinity for haeme *b* as was also demonstrated for HemQ from *L. monocytogenes* [5]. Thus disruption of the proximal H-bond network represents an important cornerstone in the interconversion of a functional Cld to a HemQ-like protein.

Cld-like proteins in Gram-positive bacteria are proposed to act as coprohaeme decarboxylases in haeme biosynthesis and potentially as regulatory haeme sensing proteins and are therefore named HemQs [3–5,7]. For both of these proposed functions the absence of the Cld-typical proximal H-bonding network is a prerequisite, since decarboxylation of a large substrate (coprohaeme) and release of the reaction product haeme *b* needs reversible binding. This work has highlighted that the CDE superfamily fold (two ferredoxin-like domains) represents a very versatile scaffold in which evolution, by inducing only a few mutations, has been able to convert a haeme sensing protein into highly efficient enzymes, either Clds or DyPs.

## Abbreviations

5c: 5-coordinated
6c: 6-coordinated
BsHemQ: HemQ from *Bacillus subtilis*
Cld: chlorite dismutase
CT1: long wavelength (>600 nm) porphyrin-to-metal charge transfer band
DaCld: Cld from *Dechloromonas aromatica*
DSC: differential scanning calorimetry
DyP: dye-decolourizing peroxidase
EPR: electron paramagnetic resonance
HemQ: Cld-like enzyme in Gram-positive bacteria
HS: high-spin
LmHemQ: HemQ from *Listeria monocytogenes*
LS: low-spin
NdCld: Cld from ‘*Candidatus* Nitrospiradefluvii’
RR: resonance Raman
SaHemQ: HemQ from *Staphylococcus aureus*
TtCld: Cld-like protein from *Thermus thermophilus*
